# Rapid design of transgene‐free cabbage with desired anthocyanin contents via HI‐Edit

**DOI:** 10.1111/jipb.13943

**Published:** 2025-06-05

**Authors:** Hongrun Li, Jiaming Shen, Xinyu Zhao, Jialei Ji, Yong Wang, Limei Yang, Mu Zhuang, Liwang Liu, Yangyong Zhang, Honghao Lv

**Affiliations:** ^1^ State Key Laboratory of Vegetable Biobreeding, Institute of Vegetables and Flowers Chinese Academy of Agricultural Sciences Beijing 100096 China; ^2^ College of Horticulture Nanjing Agricultural University Nanjing 210095 China

## Abstract

The HI‐Edit system combines haploid induction and CRISPR/Cas‐based genome editing to provide a promising way to design crops with desired traits in a rapid, precise and transgene‐free manner. HI‐Edit was applied to produce cabbages with desired anthocyanin contents.

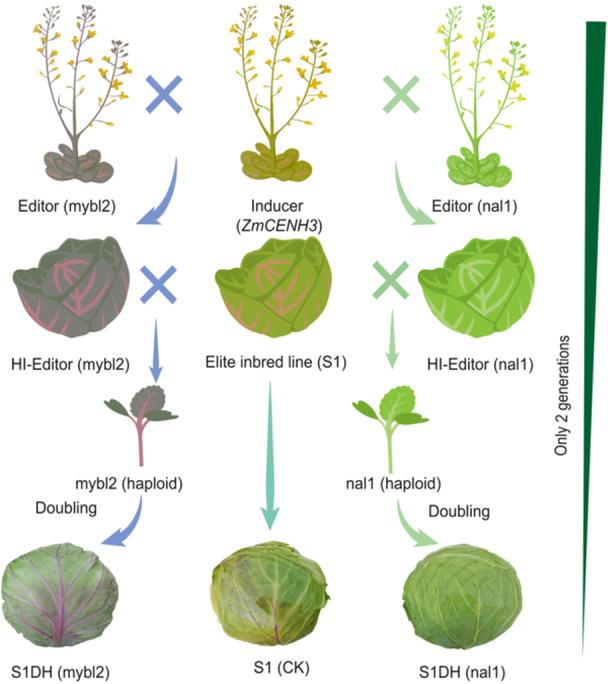

Global food production is a major challenge currently facing humanity, and the primary way to secure global food security is to increase crop yields and enhance nutritional value. Heterosis utilization by crossing of two different homozygous inbred lines has long been applied to increase crop yield. Doubled haploid (DH) technology greatly shortens the breeding process of homozygous lines by up to 3–5 years. In recent years, new haploid induction (HI) techniques based on genes such as DOMAIN OF UNKNOWN FUNCTION 679 membrane protein (*DMP*) and *CENTROMERIC HISTONE3* (*CENH3*) were exciting progresses in generating DHs in a more efficient and simple way (Jiang et al., 2022; Qu et al., 2024). Further, the clustered regularly interspaced short palindromic repeats (CRISPR)/CRISPR‐associated protein (Cas) technique has been proven to be flexible and precise in the generation of crops with desired traits. For example, enhancement of key nutrients such as carotenoids and anthocyanins via CRISPR/Cas9 has been reported in field crops such as rice (Dong et al., 2020).

The design of modern crops requires the improvement of key traits in a rapid and transgene‐free manner. One promising approach is to combine HI and CRISPR/Cas, which is called HI edit technology (HI‐Edit) (Kelliher et al., 2019). This technology has been used in maize to rapidly and precisely edit the gene *ZmLG1* of elite lines that are resistant to transformation (Wang et al., 2019). However, this technique has not been reported in a dicotyledonous crop species despite its demonstration in the model species Arabidopsis (Kelliher et al., 2019).

Vegetable crops provide humans with abundant nutrients and health‐promoting metabolites. For example, cole crops such as cabbage, which are members of *Brassica*, are planted globally and provide minerals, vitamins, and beneficial ingredients such as anthocyanins. On the market, two new types of cabbages with different levels of anthocyanins content are needed: one is completely green cabbage without any anthocyanins, and the other is a salad type that tastes crisp and tender with increased anthocyanins and light purple color, which is different from the traditional red cabbage with coarse and hard texture. This report describes studies aimed at designing these two types of cabbages rapidly via HI‐Edit. First, we chose the *CENH3* gene to develop a haploid inducer (Han et al., 2024). Second, *NAL1* (a structural gene involved in anthocyanin synthesis and encoding a dihydro‐flavonol‐4‐reductase (DFR)‐like protein) and *MYBL2* (a gene encoding an R3‐MYB transcription factor that negatively regulates anthocyanin synthesis) were selected as the editing targets (Figure 1G; Dubos et al., 2008; Yuan et al., 2023) in cabbage S1, a founder parental line with crisp and tender taste but accumulates a small amount of anthocyanins in the outer leaves, which affects the appearance quality of the derived hybrids.

**Figure 1:**
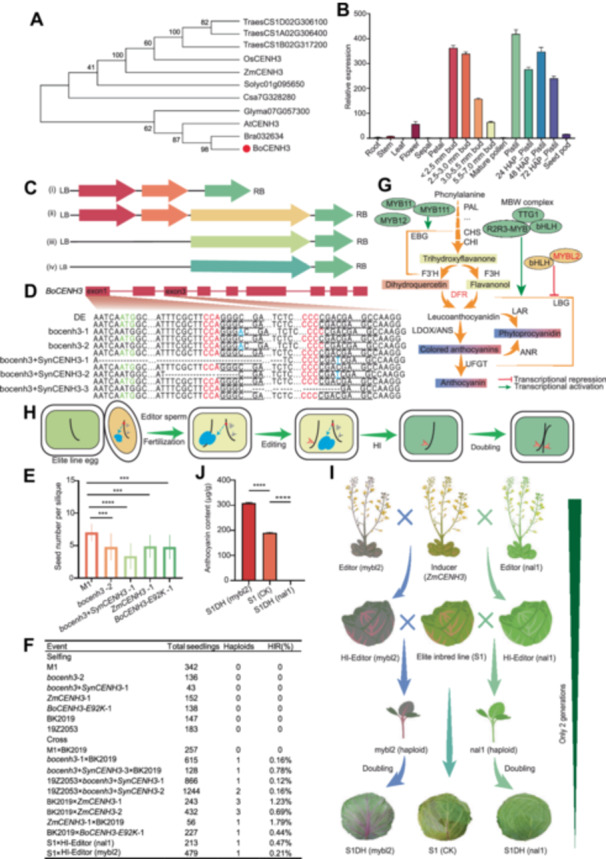
Haploid induction edit technology (Hi‐Edit)‐based rapid design of cabbage with customized anthocyanin contents **(A)** Phylogenetic analysis of *CENH3* homologs in nine species. *BoCENH3* is indicated in red. A neighbor‐joining phylogenetic tree (1,000 bootstrap replications) was constructed via MEGA7 software. **(B)** Relative expression of *BoCENH3* in cabbage tissues. HAP, hours after pollination. Three biological replicates were performed. Error bars: means ± *SD*s. **(C)** Schematic diagram of the *CENH3* constructs. (i) the clustered regularly interspaced short palindromic repeats (CRISPR)/CRISPR‐associated protein 9 (Cas9) knockout vector targeting *BoCENH3*. AtU6:sgRNA represents single‐guide RNA expression driven by the Arabidopsis U6‐26 promoter; 35S:Cas9, represents Cas9 expression driven by the CaMV 35S promoter; Bar: bialaphos resistance gene. (ii) ProBoCENH3, *BoCENH3* DNA coding sequence (CDS) represents the transfer of the synonymous point mutation CDS of *BoCENH3* driven by a native promoter. (iii) Transferring a maize *ZmCENH3*. (iv) Introducing a *BoCENH3* mutant with the E92K mutation. **(D)** Gene sequences of lines *bocenh3* and *bocenh3+SynCENH3*. Target sequences underlined; insertions in blue; translation starting site ATG in green; deletions marked as ‘‐’ and protospacer‐adjacent motif (PAM) sequences in red. **(E)** Seed number per silique of M1, *bocenh3‐2*, *bocenh3+SynCENH3*‐1, *ZmCENH3*‐1, *BoCENH3‐E92K*‐1. Error bars: means ± SDs. *n* = 15; asterisks: significant differences (***P < 0.001; ****P < 0.0001; Student's *t* test). **(F)** Haploid induction rate (HIR) of the inducer lines determined by selfing and crossing. **(G)** Anthocyanin biosynthesis and regulation. Key enzymes: PAL, CHS, CHI, F3H, DFR, LDOX/ANS, UFGT, LAR, ANR. EBG, early biosynthesis‐related genes; LBG, late biosynthesis‐related genes. bHLH, R2R3‐MYB, and TTG1 form transcriptional MBW (MYB‐bHLH‐WDR) complexes can modulate the expression level of target genes. **(H)** Model of the HI‐Edit process. The gray arrow indicates the edited *nal1* or *mybl2* locus; the red arrow indicates the target site edited in the haploid embryo. **(I)** HI‐Edit‐based generation of cabbages with increased or eliminated anthocyanin. **(J)** Total anthocyanin contents in S1DH (mybl2), S1 (CK), and S1DH (nal1). Error bars: means ± *SD*s. *n* = 3; asterisks: significant differences (****P < 0.0001; Student's *t* test).

To assess the ability of *CENH3* misexpression for HI in *B. oleracea*, we first examined the 12 putative *CENH3*‐like genes in the cabbage genome. Among them, *BoCENH3* (*Bol018461*) sequence was highly similar to the maize gene, *ZmCENH3*, with 80% sequence similarity (Figure 1A; Tables S1–S3). Quantitative reverse‐transcription – polymerase chain reaction (RT‒PCR) analysis revealed that *BoCENH3* expressed highly in the flower buds and pistils, especially in < 2.5 mm buds and pistils 48 h after pollination (Figure 1B). We then tested the induction ability of *CENH3* via four strategies (Figure 1C): (i) knockout of *BoCENH3*; (ii) knockout of *BoCENH3* and simultaneous transfer of the *BoCENH3* DNA coding sequence (CDS) with six synonymous point mutations at the target sequence of the guide RNA (gRNA) to avoid being edited (Table S5); (iii) transfer of *ZmCENH3* (Table S6); and (iv) transfer of a nonsysnonymous mutant *BoCENH3*, a CDS with a G‐A mutation in the 274th nucleotide sequence (i.e., an E‐A mutation in 92nd amino acid sequence) (Table S7). For the first and second strategies, a CRISPR/Cas9 construct with two specific gRNAs targeting the first and third exons of *BoCENH3* was generated and introduced into cabbage line M1 (a line with high genetic transformation efficiency; Zhao et al., 2022) via Agrobacterium‐mediated transformation. For the second to fourth strategies, we chose the promoter *ProBoCENH3* (native promoter of *BoCENH3*) to drive the genes (Table S4). The edited lines of the four strategies were confirmed by sequencing or marker testing and were named *bocenh3*, *bocenh3*+*SynCENH3*, *ZmCENH3*, and *BoCENH3‐E92K*, respectively (Figure 1D). Compared with M1, the edited lines of the four strategies were dwarf, and the leaves were crinkled (Figure S1) with all the lines fertile. After selfing, the seed setting rates of the edited lines significantly decreased (Figure 1E).

To evaluate the HI rate (HIR) of the T_1_ generation of the four types of inducers, we crossed them with BK2019 (a test kale line; Yuan et al., 2023) and 19Z2053 (a test cabbage line; Zhao et al., 2022). Among the progenies, the haploids were similar to those of the test lines BK2019 or 19Z2053 but presented smaller flowers, male sterility and dwarfed plants (Figure S2), which was further confirmed by flow cytometry analysis and molecular marker analysis (Figures S3, S4). The results revealed that *CENH3* misexpression can induce haploids as both male and female parents, and the HIRs were 0.12%–1.79% for all the crosses (Figure 1F). Among them, the inducer *ZmCENH3*‐1 presented the highest HIR of 1.23%–1.79% in the reciprocal crosses; the inducer *bocenh3*+*SynCENH3* presented an HIR of 0.12%–0.78%; and the inducers *bocenh3* and *BoCENH3‐E92K* presented relatively low HIRs. Additionally, the homozygous *ZmCENH3‐1* individuals of the T_1_ generation were selected for further HI‐Edit tests as inducers.

To construct the HI‐Edit system, we first generated *nal1* and *myl2* mutants with cabbage line M1 via CRISPR/Cas9 (Figure 1G), and the individuals of T_1_ with CRISPR/Cas9 components were kept as editors (nal1) with a complete green phenotype and editors (mybl2) with a light purple phenotype. The two editors were subsequently crossed with the inducer *ZmCENH3‐1*. The plants of the selfing progenies carrying *ZmCENH3‐1* and CRISPR/Cas9 cassettes, with *NAL1* and *MYBL2* being edited, were selected as the HI‐Editor (nal1) and HI‐Editor (mybl2), respectively (Figure 1H, I). The two HI‐Editors were then crossed with the elite inbred line S1 to test their ability of inducing haploids with the target genes being edited. A total of 104 individuals of 213 obtained from the cross S1×HI‐Editor (nal1) were completely green, whereas one progeny was haploid, corresponding to a HIR of 0.47%. A total of 245 of the 479 individuals obtained from the cross S1×HI‐Editor (mybl2) were purple, whereas one of the 245 progenies was haploid, corresponding to a HIR of 0.21% (Figure 1F). The two haploids were doubled, and the anthocyanin contents of the leaves were measured via spectrophotometry. The results revealed that the anthocyanin contents of S1DH (mybl2), S1 and S1DH (nal1) were 308, 189, and 0 μg/g, respectively, indicating that *NAL1* and *MYBL2* of line S1 were successfully edited by HI‐Editors (Figure 1J) with absence of Cas9 (Figure S5), as confirmed by sequencing (Figures S6, S7). The DH lines could be directly applied in the breeding of market‐oriented cabbages with high or no anthocyanins.

In summary, we developed a HI‐Edit system to design cabbages with desired anthocyanin contents. This HI‐Edit system is the first application case in dicotyledonous crops, which combines conventional breeding with modern genetic engineering techniques of HI and genome editing. In addition, unlike other HI systems, especially the *DMP*‐based HI system in *B. oleracea* (Zhao et al., 2022), this system can induce haploids as both male and female parents *in vivo*. Additionally, compared with the reported *CENH3*‐based HI system in broccoli (Han et al., 2024), we supplied two additional ways to edit CENH3, with the HIR increasing slightly from 1.29% to 1.79%. In the future, other specific target genes, such as broad‐spectrum disease resistance or stress tolerance genes, can be selected in combination with new editing techniques, such as prime editing and base editing. Furthermore, other elite inbred lines can be used to test the ability of inducing haploids with the target genes edited for higher HI‐Edit percentage. This HI‐Edit system holds great potential for accelerating the progress of crop breeding.

## CONFLICTS OF INTEREST

The authors declare they have no conflicts of interest.

## AUTHOR CONTRIBUTIONS

H.Lv. designed the work. H.Li., X.Z. and J.S. performed the experiments. H.Li., and H.Lv. wrote and revised the manuscript. J.J., Y.W., L.Y., M.Z., L.L. and Y.Z. analyzed the data and revised the manuscript. All authors have read and approved the final manuscript.

## Supporting information

Additional Supporting Information may be found online in the supporting information tab for this article: http://onlinelibrary.wiley.com/doi/10.1111/jipb.13943/suppinfo

**Figure S1.** The characteristics of M1 and edit lines of four strategies
**Figure S2.** The characteristics of haploid and diploid
**Figure S3.** Molecular markers analysis of haploid and diploid
**Figure S4.** Flow cytometry analysis of haploid and diploid plants
**Figure S5.** Detection of Cas9 in haploids
**Figure S6.** Mutations at the target region of S1DH (mybl2)
**Figure S7.** Mutations at the target region of S1DH (nal1)
**Table S1.** Full sequence of *BoCENH3*
**Table S2.** Coding sequence of *BoCENH3*
**Table S3.** Amino acid sequence of BoCENH3
**Table S4.** Sequence of the promoter *ProBoCENH3*
**Table S5.** Sequence of *SynCENH3* DNA coding sequence (CDS) sequence
**Table S6.** Sequence of *ZmCENH3* DNA coding sequence (CDS) sequence
**Table S7.** Sequence of *BoCENH3‐E92K* sequence
**Table S8.** The InDel marker primers between 19Z2053 and S1
**Table S9.** The raw data of the anthocyanin measurements
