# Supplementary material for: Rapid design of transgene‐free cabbage with desired anthocyanin contents via HI‐Edit
Source: J Integr Plant Biol. 2025 Jun 5;67(9):2259–61. doi: 10.1111/jipb.13943 (PMC12402743; doi:10.1111/jipb.13943)
Supplement: Supplementary file 1 — Figure S1. The characteristics of M1 and edit lines of four strategies Figure S2. The characteristics of haploid and diploid Figure S3. Molecular markers analysis of haploid and diploid Figure S4. Flow cytometry analysis of haploid and diploid plants Figure S5. Detection of Cas9 in haploids Figure S6. Mutations at the target region of S1DH (mybl2) Figure S7. Mutations at the target region of S1DH (nal1) Table S1. Full sequence of BoCENH3 Table S2. Coding sequence of BoCENH3 Table S3. Amino acid sequence of BoCENH3 Table S4. Sequence of the promoter ProBoCENH3 Table S5. Sequence of SynCENH3 DNA coding sequence (CDS) sequence Table S6. Sequence of ZmCENH3 DNA coding sequence (CDS) sequence Table S7. Sequence of BoCENH3‐E92K sequence Table S8. The InDel marker primers between 19Z2053 and S1 Table S9. The raw data of the anthocyanin measurements [file JIPB-67-2259-s001.docx]

**Table S1**. Full sequence of *BoCENH3*.

|  | Sequence |
| --- | --- |
| *BoCENH3* | ATGGCGAGAACCAAACATTTCGCTTCCAGGGCACGAGATCGCAATCGAACTAGTTAGTACTCTCTCTCTCTCTGCCTTTTTTTTGATATTTATTTTCTAGGTTAAACCCTAATTTGGCATCTGAAATTTGTAGATGCGACTGCTTCATCTTCGGCGGCGGCGGCGGAAGGTCCGAGTGCGGTACGTCATCTATTTTCTTTTCCCGTTTTAGGTTTTTACGCAAATCTCGTTACTGTTTTTTTGACGAATCGATTGAAATGTGTAGACCCCGACGAGAAGAGAAGGCAGCCAAGGAGAAGCTCAACAGAGTGAGTCTTTCTATTTCATTTTCTGAGATCCATGAATCCTTTTCATCTCTCGTGTGTTGTGACATGAATCAATTGCAGCAGCAACTCCTACTACGACTCCACCAGCCGGTAGAAAAGTAAGTTACATTTCCATTTCACACCATTCATTTGCTTCTTTATCAACAAACTGCTCTCTCATCTGTTTTTTTTGTTTTGTTTTGGTTTTGTGAAGAAAGGAGGGACTAAGCGAACTAAACAAGCTATGCCTAAAAGTTAGTGACAGATTTTAAAATCTCTATTTTGGATCATCATTCTCTCAGGACATGTCTATTTGCATTTGTTCTTATTATGTCTGTCTGTCTGTCTTTGTCCCCCTTGTAGGTTCCAACAAGAAGAAGACATTCCGTTACAAGCCTGGAACCGTTGCCCTCAGAGAGATTCGCCATTTCCAGAAGACCACCAAACTTCTTATCCCTGCCGCTAGTTTCATCCGAGAAGTTAGTAATGAACTTTGTTATTCATACATTCCCGCTTACTTGTTTTCAATGACTCTGCAATTACTGATATAGAATTTGGAGCAACCATTATGGGGTGATTTCTCTAACTACAAATTACTAATACTATCCCAGGTGAGAAGTGTCACCCAGATCTTTGCCCCTCCCGATGTTACCCGTTGGACTGCTGAAGCTCTTATGGCTATTCAAGAGGTACGTGTACTCCTTCCCTCTTTTGTTTCCTATTTTCCACTTGATGTCTAATTTAAACTGATCGTTTTTTTTTTATATTTCTTTTGGTGTGGGGCGGGGCAGGCGGCTGAAGATTTTTTAATTGGCTTGTTCTCTGATGCTATGCTTTGCGCTATCCATGCAAGGCGTGTTACTCTAAGTAAGTAGTACTCCCCAAAATAAGGAAACCCATTTTATATACAACATTGCCTCATCCATGTCTGCTTCTCTTCATATCAGTGAGAAAAGATTTTGAGCTTGCACGCCGTCTTGGAGGAAAAGGCAGACCATTGTGA |

**Table S2**. Coding sequence of *BoCENH3*. The red regions are the target sequences.

|  | Sequence |
| --- | --- |
| *BoCENH3-CDS* | ATGGCGAGAACCAAACATTTCGCTTCCAGGGCACGAGATCGCAATCGAACTAATGCGACTGCTTCATCTTCGGCGGCGGCGGCGGAAGGTCCGAGTGCGACCCCGACGAGAAGAGAAGGCAGCCAAGGAGAAGCTCAACAGACAGCAACTCCTACTACGACTCCACCAGCCGGTAGAAAAAAAGGAGGGACTAAGCGAACTAAACAAGCTATGCCTAAAAGTTCCAACAAGAAGAAGACATTCCGTTACAAGCCTGGAACCGTTGCCCTCAGAGAGATTCGCCATTTCCAGAAGACCACCAAACTTCTTATCCCTGCCGCTAGTTTCATCCGAGAAGTGAGAAGTGTCACCCAGATCTTTGCCCCTCCCGATGTTACCCGTTGGACTGCTGAAGCTCTTATGGCTATTCAAGAGGCGGCTGAAGATTTTTTAATTGGCTTGTTCTCTGATGCTATGCTTTGCGCTATCCATGCAAGGCGTGTTACTCTAATGAGAAAAGATTTTGAGCTTGCACGCCGTCTTGGAGGAAAAGGCAGACCATTGTGA |

**Table S3** Amino acid sequence of *BoCENH3.*

|  | Amino acid sequence |
| --- | --- |
| *BoCENH3* | MARTKHFASRARDRNRTNATASSSAAAAEGPSATPTRREGSQGEAQQTATPTTTPPAGRKKGGTKRTKQAMPKSSNKKKTFRYKPGTVALREIRHFQKTTKLLIPAASFIREVRSVTQIFAPPDVTRWTAEALMAIQEAAEDFLIGLFSDAMLCAIHARRVTLMRKDFELARRLGGKGRPL |

**Table S4** Sequence of the promoter *ProBoCENH3*.

|  | Sequence |
| --- | --- |
| *ProBoCENH3* | TGTGGACATTACACGCAGATCGTGTGGAGAGATAGCACGAAGGTTGGGTGTGCACGTGTGGATTGCTCAAATGGTGGCCTTTATGCGATTTGCGTTTATAATCCACCGGGAAATTACGAAGGTGAGAACCCGTTTGGAAATTATGAAGACCAGATTGGTCTTGTCCGGGAGGATCCACCGGCGGTGGTCGTTACTCCATTTTAACTTGATGTCTTGAAAGCAGAGGACATGGTATGGTGGCGGCAGAATCTATTTTTAGTTGTTAATTTTTCTTTTCCTCTGATTCTTTTTATTTTTTTCGAATGAACTAACTTTGGGTTTATTCAGAAGAATTATCATCTAAAAACTGATTCAATAAACAAAATAATTTACATATTTCACAATGAGCCATTAGTAAACAAGTCGAAAGTGAAACCAAATGGGAAGAGAACAATTTTAATAAAAATATGTTCTAATTTCCTACTTTTTATGAATTGAACTCCCGAAGAGAATGGCCGAAGAACGGAGTAAAAGCTCAATGATTGTAAAAGCTATGTATTCTCTTGCTTTGAGGAAAAAGCTTTTTGTTTGCACTCATAGGCCTGATATGTTGTGATGGCTCTTTACATATTGGGTCTTTTGTGGTCTATTAAACGGTTACTGAAGAATTAGTTTATCGCATTTAAAAAAAAATATTTGAAAACCACATATCTAAAATCTCAATATATTATTTATTGATAGATGAAAAGAAAAATAGTTTATAAAATAATATTAATTGGTAAATGGAAGTCAAAATTTTACATTTAATACATGACTAGTTCGATTCTCCGGCTGAAACCCATTTACCACTGAGCCAAGACCACTTGATTATATATATCAAGCTCTTGTTTTTTGCATTAACAAAAGTTGCATCCAAAATTTCAAAACAACAACTAAATTAATGTCTTTCTATTTTCAAAGTTTTATCTCCAAACCTATTAATAGTTTAATTTTTTTTTTTAAAATTAGGAAACCGGTTAAAATATATTTTAAATACAAAAAACTTAAACAATGAATCATTTATTTATTTATTCTTCAAAATTTAAATATCCGAACCCGGCCCAAAATATCCGAACCCGAACATAAAATATCCGAACCCGACTCGAAGTGTAGAAAATATCCGAACGGGTTTTATACCTTTATACTGAAATACCCTATACGAACCCGAATGTGTATCCGAACGCCCCCTACAATATATGATCATCATTTGTATCTTGATTGAACAAAAAAAAAGTTAAACTATTGATCACAAAATTTTCAATGTGAGACTTTTACCATTTTTAGTCATTTATAGTCGTTTTTAAAAATTCAAAATGTAACTTATAAGAAAAAATCTAATTTTTTTTATTATATGCTTAATGTGATTGTTTAATTTCTTTTAATAATATAAAATTAAACAAAAAATGAGAGGTTAAAAAAATTGTTATCAAATATGTATTATTCATAATCATTAATTGTCATATATATGTTAATTATATTAGGTAATTTCGTAGTTTTTATTTAAGAAAAGAAAAAAATATTATTTTGTACACTACTAATTAATTTGATAGTTAGTTTAATAAAAAATATATTATATTATTATATGGACCAACTTATTTTTCTAAAAAAAAACCACTGTTTTAAAAACCAAACCAACTATAAACCGGAGATATACCGGATTGAGTGGCTAAAACACTCTTTGTATATATGTGCTGAGCAAACCCTCTGAGTGAGATGGCGTGTTAAGAAGTAGGAGGACCATTCATGCCTCTTATGAGTTGTAGTCTGTGTGTACAAAAAAGAAGCGTTGGTGTGAAAGAAAGCAGAAGGATTTGAAAATCAAAAAAATTGAAGGAGAAGCGGGAAAACAAATAATCTCTCCCTCCGCTTTTTTTTCTCCAAATAATCAATCTCTCATTTCATTTGTTAACCCAAGTTTTTGATAATTATTTCAAAGGGGTTTATTTATCTT |

**Table S5** Sequence of *SynCENH3* CDS sequence. Red regions are the targets and yellow highlights are synonymous point mutations.

|  | Sequence |
| --- | --- |
| *SynCENH3* CDS | ATGGCGAGAACCAAACATTTCGCTTCAAGAGCGCGAGATCGCAATCGAACTAATGCGACTGCTTCATCTTCGGCGGCGGCGGCGGAAGGTCCGAGTGCGACACCAACAAGAAGAGAAGGCAGCCAAGGAGAAGCTCAACAGACAGCAACTCCTACTACGACTCCACCAGCCGGTAGAAAAAAAGGAGGGACTAAGCGAACTAAACAAGCTATGCCTAAAAGTTCCAACAAGAAGAAGACATTCCGTTACAAGCCTGGAACCGTTGCCCTCAGAGAGATTCGCCATTTCCAGAAGACCACCAAACTTCTTATCCCTGCCGCTAGTTTCATCCGAGAAGTGAGAAGTGTCACCCAGATCTTTGCCCCTCCCGATGTTACCCGTTGGACTGCTGAAGCTCTTATGGCTATTCAAGAGGCGGCTGAAGATTTTTTAATTGGCTTGTTCTCTGATGCTATGCTTTGCGCTATCCATGCAAGGCGTGTTACTCTAATGAGAAAAGATTTTGAGCTTGCACGCCGTCTTGGAGGAAAAGGCAGACCATTGTGA |

**Table S6** Sequence of *ZmCENH3* CDS sequence.

|  | Sequence |
| --- | --- |
| *ZmCENH3 CDS* | ACCATCTCCTGCCCCCTTAAAAAAAAGACTCACCGTCGACACGCCCTCCCGTCCCGAGAGTTCTGAATCGAAACCGTCGGCCACGAGAGCAGTGCGAGGCGCCCACCGCGATGGCTCGAACCAAGCACCAGGCCGTGAGGAAGACGGCGGAGAAGCCCAAGAAGAAGCTCCAGTTCGAGCGCTCAGGTGGTGCGAGTACCTCGGCGACGCCGGAAAGGGCTGCTGGGACCGGGGGAAGAGCGGCGTCTGGAGGTGACTCAGTTAAGAAGACGAAACCACGCCACCGCTGGCGGCCAGGGACTGTAGCGCTGCGGGAGATCAGGAAGTACCAGAAGTCCACTGAACCGCTCATCCCCTTTGCGCCTTTCGTCCGTGTGGTGAGGGAGTTAACCAATTTCGTAACAAACGGGAAAGTAGAGCGCTATACCGCAGAAGCCCTCCTTGCGCTGCAAGAGGCAGCAGAATTCCACTTGATAGAACTGTTTGAAATGGCGAATCTGTGTGCCATCCATGCCAAGCGTGTCACAATCATGCAAAAGGACATACAACTTGCAAGGCGTATCGGAGGAAGGCGTTGGGCATGATATATAATATCCATTCTGATTGCATCATTCTTGTGAATTTGTTTGTAGGAGCTAGACATTAGTGTTGTTGAATGCTGCATGGTTCCTAATCCTTTTCGCAGTCTAACATCTGTGGAGTTAGTATGTTACATGGCAACAGCTGAACATCTGTGGACTATA |

**Table S7** Sequence of *BoCENH3-E92K* sequence. The yellow highlight is the E92K point mutation, which means the 92nd amino acid glutamic acid is mutated to lysine.

|  | Sequence |
| --- | --- |
| *BoCENH3-E92K* | ATGGCGAGAACCAAACATTTCGCTTCCAGGGCACGAGATCGCAATCGAACTAATGCGACTGCTTCATCTTCGGCGGCGGCGGCGGAAGGTCCGAGTGCGACCCCGACGAGAAGAGAAGGCAGCCAAGGAGAAGCTCAACAGACAGCAACTCCTACTACGACTCCACCAGCCGGTAGAAAAAAAGGAGGGACTAAGCGAACTAAACAAGCTATGCCTAAAAGTTCCAACAAGAAGAAGACATTCCGTTACAAGCCTGGAACCGTTGCCCTCAGAAAGATTCGCCATTTCCAGAAGACCACCAAACTTCTTATCCCTGCCGCTAGTTTCATCCGAGAAGTGAGAAGTGTCACCCAGATCTTTGCCCCTCCCGATGTTACCCGTTGGACTGCTGAAGCTCTTATGGCTATTCAAGAGGCGGCTGAAGATTTTTTAATTGGCTTGTTCTCTGATGCTATGCTTTGCGCTATCCATGCAAGGCGTGTTACTCTAATGAGAAAAGATTTTGAGCTTGCACGCCGTCTTGGAGGAAAAGGCAGACCATTGTGA |

**Table S8** The InDel marker primers between 19Z2053 and S1.

| Primer name | Forward primer sequence (5’ to 3’) | Reverse primer sequence (3’ to 5’) |
| --- | --- | --- |
| LY4 | TGTTTTGTGGTTACTTGCTTGC | GGTACCCCTCTGATCATCGG |

**Table S9** The raw data of the anthocyanin measurements.

|  | OD530 | OD620 | OD650 | ODλ | The anthocyanin level |
| --- | --- | --- | --- | --- | --- |
| S1DH (mybl2) -1 | 0.409 | 0.240 | 0.454 | 0.1476 | 307.50 |
| S1DH (mybl2) -2 | 0.39 | 0.221 | 0.421 | 0.149 | 310.42 |
| S1DH (mybl2) -3 | 0.404 | 0.238 | 0.432 | 0.1466 | 305.42 |
| S1 (CK) -1 | 0.297 | 0.188 | 0.353 | 0.0925 | 192.71 |
| S1 (CK) -2 | 0.242 | 0.14 | 0.267 | 0.0893 | 186.04 |
| S1 (CK) -3 | 0.259 | 0.153 | 0.306 | 0.0907 | 188.96 |
| S1DH (nal1) -1 | 0.079 | 0.074 | 0.124 | 0 | 0.00 |
| S1DH (nal1) -2 | 0.091 | 0.086 | 0.136 | 0 | 0.00 |
| S1DH (nal1) -3 | 0.068 | 0.063 | 0.113 | 0 | 0.00 |


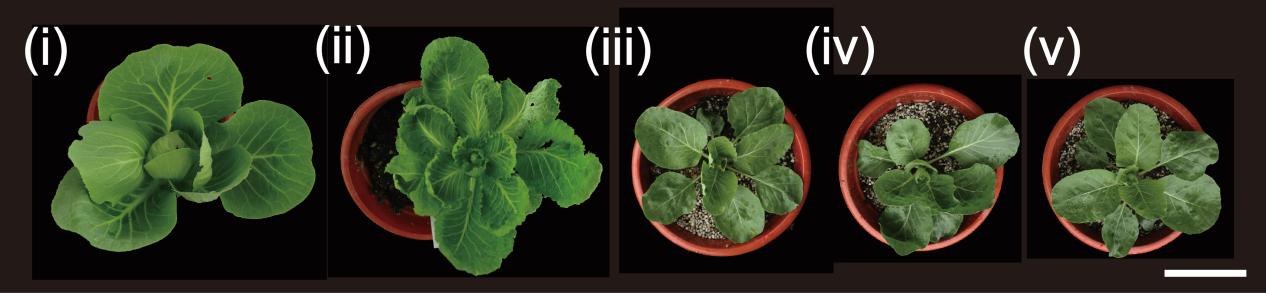


**Figure S1** The characteristics of S1 and edit lines of four strategies. (i) M1. (ii) *bocenh3-2*. (iii) *bocenh3+SynCENH3-1*. (iv) *ZmCENH3-1*. (v) *BoCENH3-E92K-1*.

Scale bars: 10 cm.


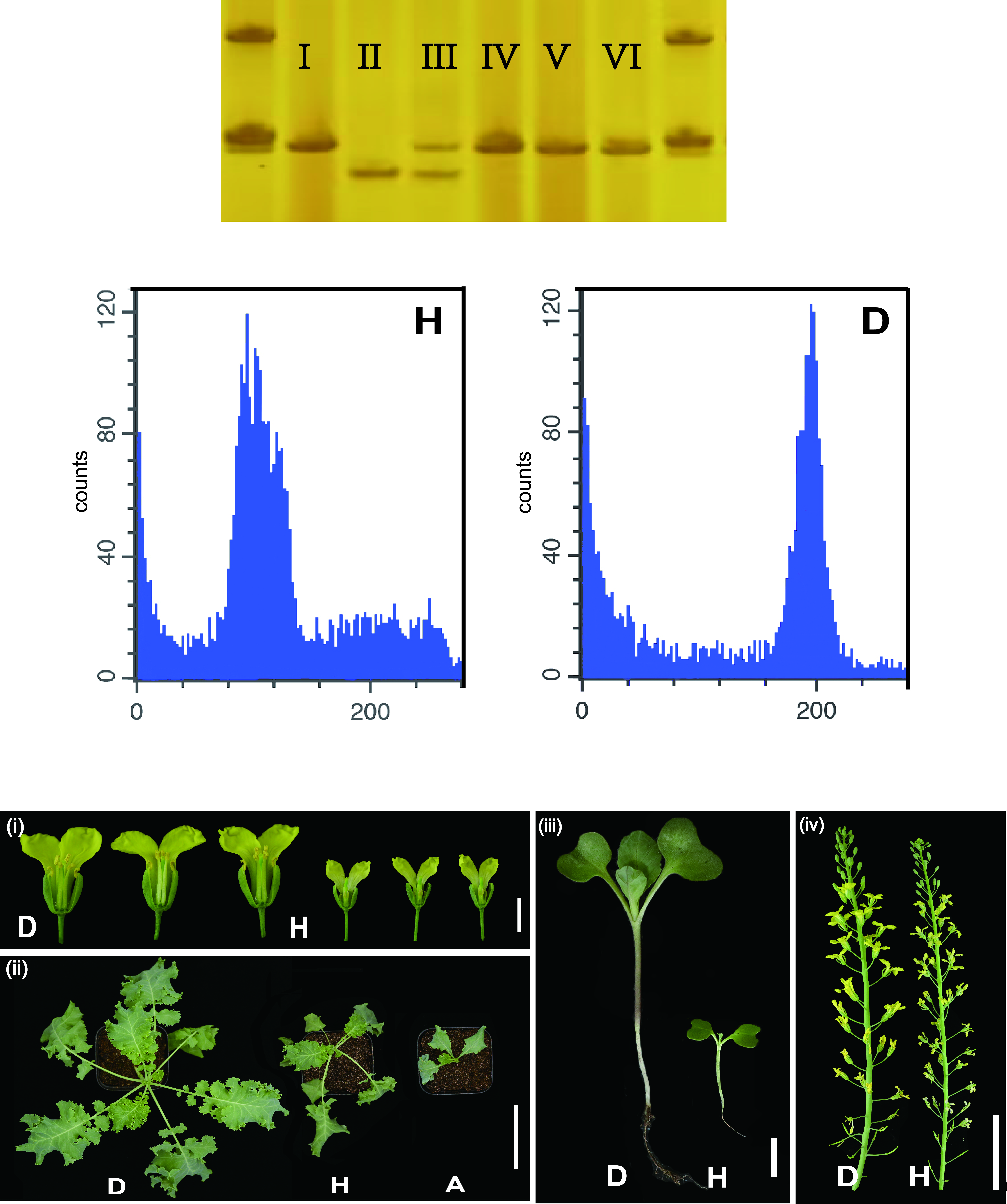


**Figure S2** The characteristics of haploid and diploid (i) Flower anatomy for haploid and diploid plants. (ii) Diploid BK2019, haploid and aneuploid from the cross *bocenh3*+*SynCENH3-3*×BK2019. (iii) Ten-day-old seedlings from the cross *bocenh3-1*×BK2019. F1 hybrid (right) and haploid (left). (iv) Flowering branch for haploid and diploid plants. H, haploid; D, diploid; A, aneuploid.

Scale bars: 1 cm (i) and (iii), 10 cm (ii) and (iv).


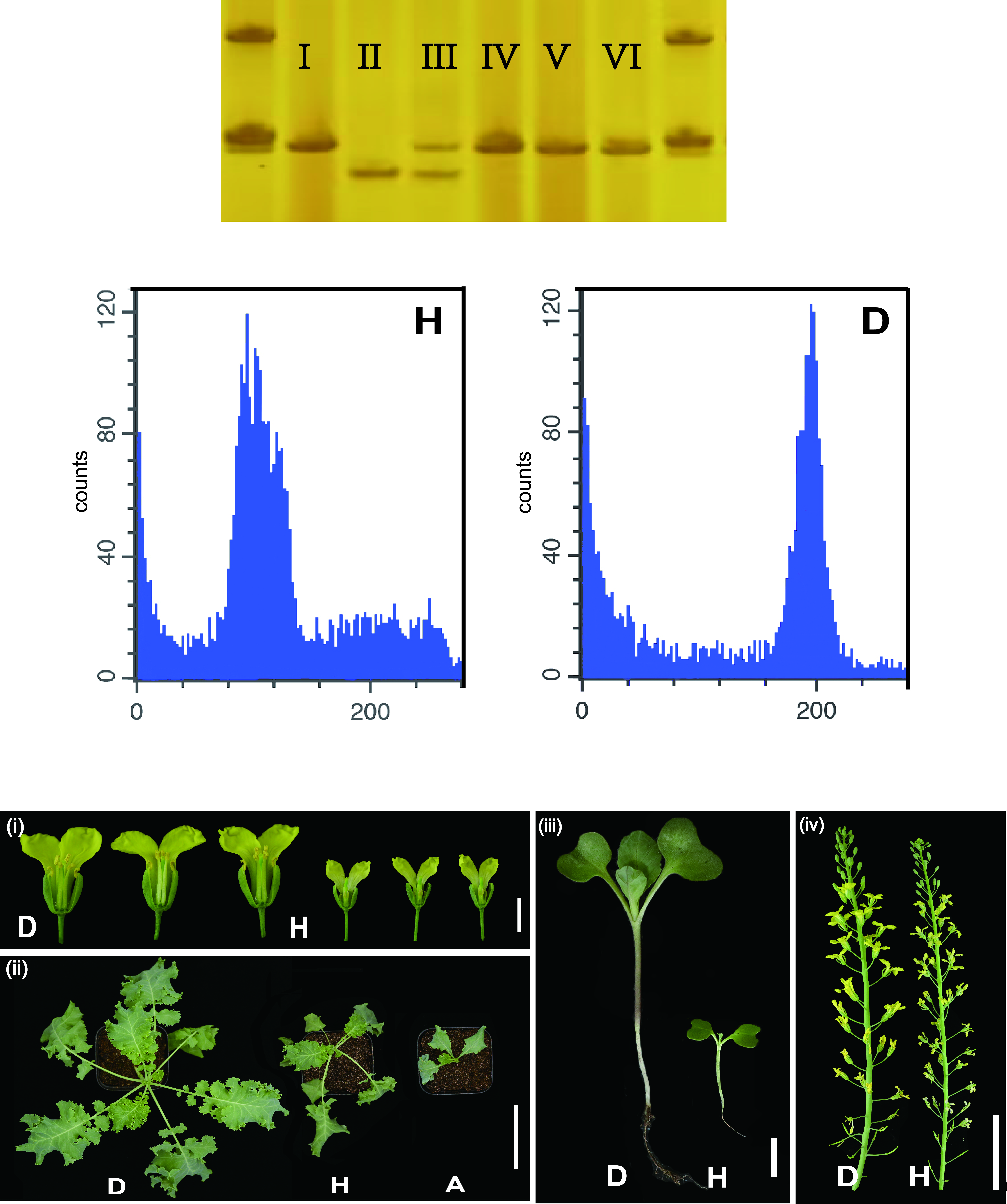


**Figure S3** Molecular markers analysis of haploid and diploid. Left and right lanes, DNA marker; I-III, PCR bands of 19Z2053, *bocenh3*+*SynCENH3-2*, and a hybrid from 19Z2053×*bocenh3*+*SynCENH3-2*, representatively; IV-VI, PCR bands of three haploids from 19Z2053.


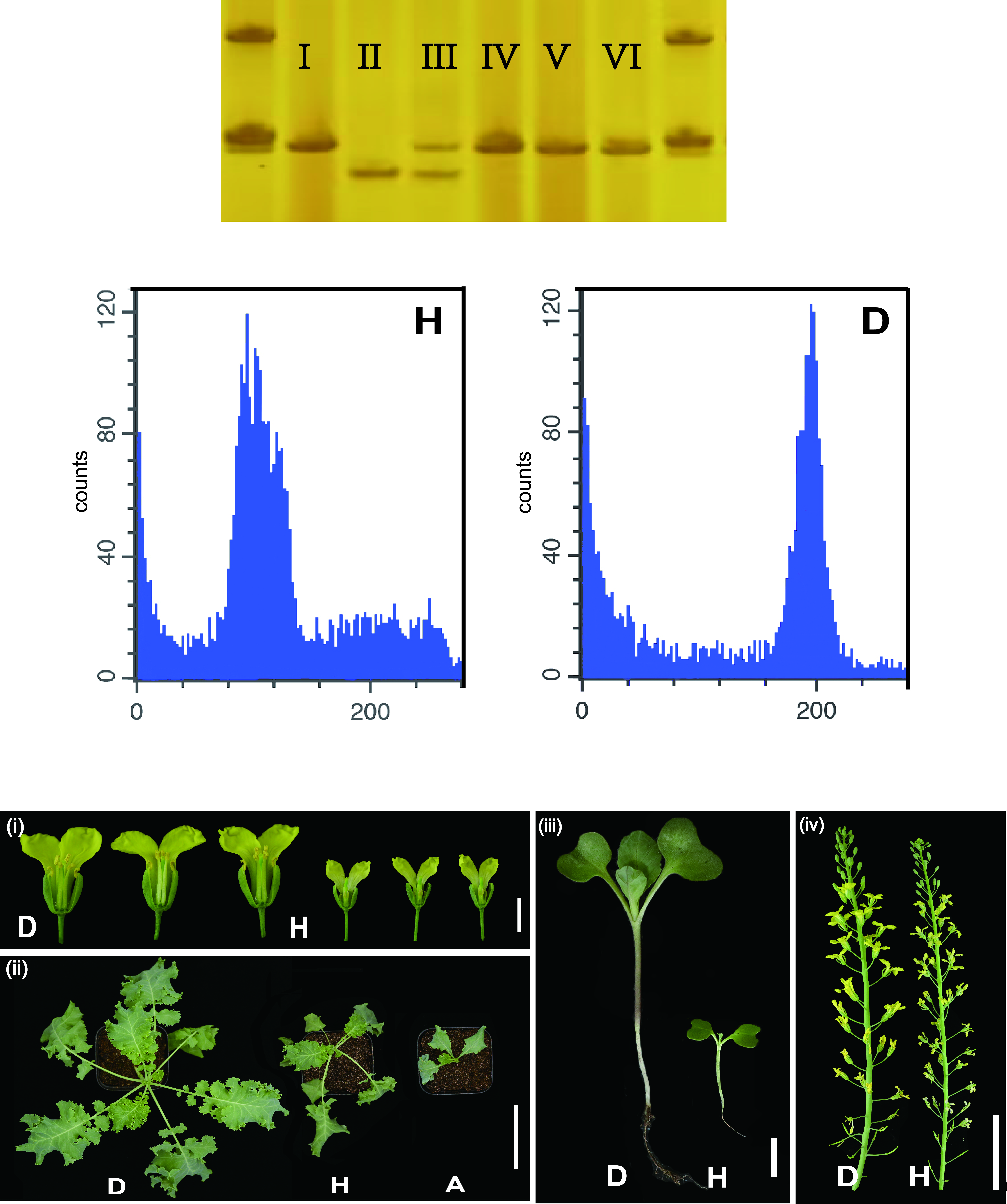


**Figure S4** Flow cytometry analysis of haploid and diploid plants. Ordinate: the number of effective cells; abscissa: DNA content; H, haploid; D, diploid.


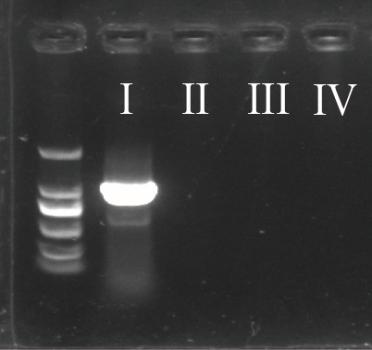


**Figure S5** Detection of Cas9 in haploids. Left lanes, DNA marker; I, positive control, II, negative Control, III, haploid from S1×HI-Editor (nal1), IV, haploid from S1×HI-Editor (mybl2).


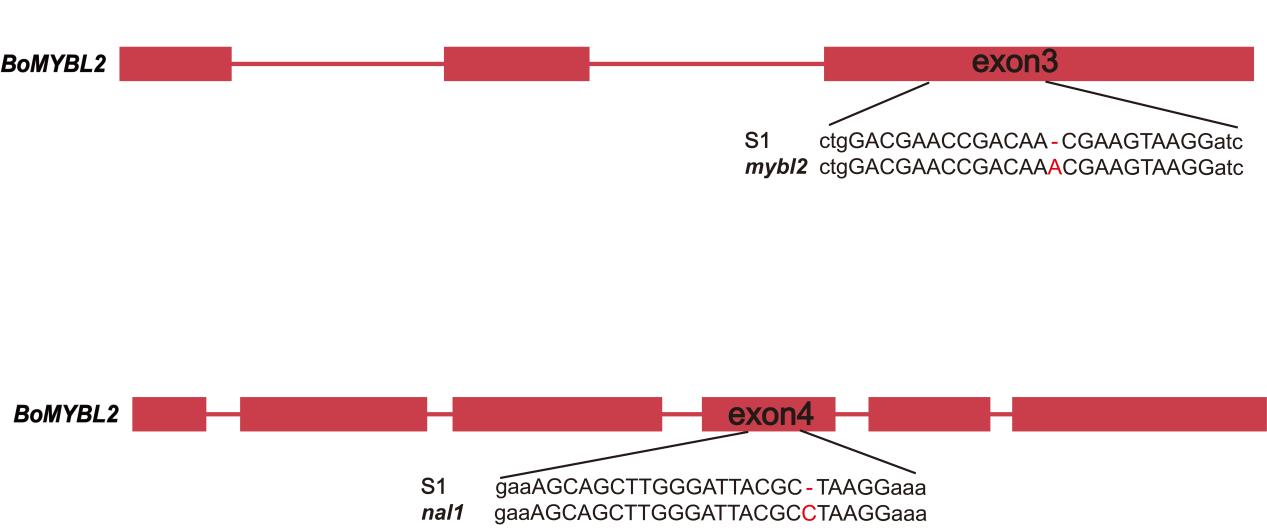


**Figure S6** Mutations at the target region of S1DH (mybl2). The mutation is highlighted in red.


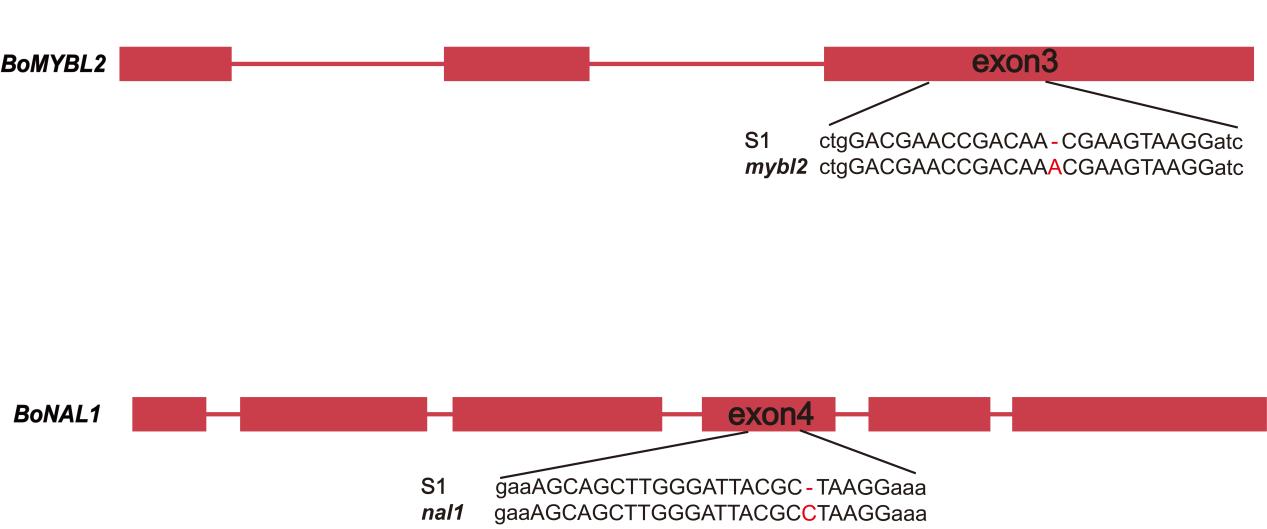


**Figure S7** Mutations at the target region of S1DH (nal1). The mutation is highlighted in red.
